# Evaluation of Thermographic Imaging in Canine Hindlimb Muscles After 6 Min of Walking—A Pilot Study

**DOI:** 10.3389/fvets.2020.00224

**Published:** 2020-04-30

**Authors:** Jennifer Repac, Leilani X. Alvarez, Ken Lamb, Robert L. Gillette

**Affiliations:** ^1^The Animal Medical Center, Integrative and Rehabilitative Medicine Department, New York, NY, United States; ^2^Lamb Statistical Consulting and Scientific Writing LLC, West Saint Paul, MN, United States; ^3^Sportsvet Veterinary Consulting Services, Lancaster, SC, United States

**Keywords:** thermography, canine, infrared thermography, exercise, 6 min walk test, muscle

## Abstract

**Objective:** To evaluate changes in superficial temperature of hindlimb muscles before and after a 6-min walk in healthy dogs.

**Methods:** Two infrared thermographic images were captured of the proximal and distal hindlimbs of 11 healthy dogs before and after a 6-min walk. Orthopedic exam and objective gait analysis confirmed the healthy status of study subjects. Superficial temperatures of the gastrocnemius, biceps femoris, and gracilis were assessed. Analysis of images was performed using 2 different methods of region of interest (ROI) selection. ROI were selected first using one point (single pixel) in the muscle and then separately by selecting a line (LN) corresponding to many points of each muscle belly from which an average was taken. *P* < 0.05 was considered significant.

**Results:** There was no significant change in temperature using point ROI before and after 6 min of walking of the gastrocnemius, gracilis, and biceps femoris muscles (*p* = 0.273, *p* = 0.349, *p* = 0.351, respectively). Using linear ROI, both biceps femoris and gracilis muscles exhibited significant increases in temperature (*p* < 0.0001, *p* = 0.032, respectively). There was no significant increase in temperature of gastrocnemius muscle for both point and linear ROI selection (*p* = 0.273, *p* = 0.448, respectively). The right biceps femoris temperatures were higher compared to left biceps femoris using the linear ROI before and after walks (*p* < 0.0001). The overall (left and right limbs pooled) standard deviation of point selected values were greater than LN selected values of the biceps femoris (1.35 and 1.11) and gastrocnemius (1.51 and 1.23). In contrast, standard deviation for the gracilis measurements were decreased using point selection vs. LN selection (1.09 and 1.3).

**Conclusions:** The biceps femoris and gracilis muscles demonstrated significant increases in surface temperature after 6 min of walking using the linear method of ROI. Measurement of numerous points along the entire length of the biceps femoris and gastrocnemius muscles may provide a more accurate assessment of the increased vascularity within the tissues resulting from work compared to single point selection.

**Clinical Significance:** Prior activity and ROI selection method should be considered when interpreting thermography results.

## Introduction

Thermography is a non-invasive tool that can be used to screen for early stages of disease, support physical exam findings, or monitor response to therapy by measuring radiant heat emitted from the skin's surface (1). Thermographic imaging has been utilized in human and equine medicine since the 1960s (2). Current applications in human medicine include breast cancer screening, assessment of osteoarthritis (3, 4), monitoring of healing after burns (5), and muscle injury (6, 7). In equine patients, thermography has been used in early detection of laminitis, tendon and ligament injuries, and thoracolumbar pain (8). In cats, thermography has been used in detection of pain (9), aortic thromboembolism (10), and hyperthyroidism (11).

There are several studies on the use of thermal imaging in canines. The ability to obtain consistent thermographic images in healthy dogs has been demonstrated (12, 13). Thermal imaging has been shown to be useful in detection of several pathological conditions in dogs including osteosarcoma (14), elbow dysplasia (15), cranial cruciate ligament rupture (16, 17), and intervertebral disc disease (18). Thermography has also been shown to have applications for canine gait analysis (19, 20).

There have been several previous studies investigating the effect of exercise on thermal images in dogs (21–23). One study, in post-racing Greyhounds, showed that the gastrocnemius muscle had a statistically significant increase in superficial temperature compared to baseline (23). Another study in military dogs trotting and running for 12 min, resulted in an increase in temperature of the biceps femoris muscle (21).

Previous studies have shown that the muscles of the hindlimb contribute to the propulsive forces during all gaits (24). One study suggested that relative dominance of the gastrocnemius muscle may play a role in cranial cruciate ligament disease and that monitoring of the gastrocnemius could play a role in injury prevention and rehabilitation (25).

It has previously been shown that the 6-min walk test [6MWT; (26)] can effectively differentiate healthy dogs from dogs with congestive heart failure (27), pulmonary disease (28), and neuromuscular disease (29, 30). The 6MWT has also been utilized as a tool for monitoring changes in cardiopulmonary function over time (31, 32). The ability of the 6MWT to differentiate individuals with mild cardiopulmonary disease suggests an increase in oxygen demand and work from the muscles required for locomotion. However, the thermographic effects of the 6MWT on hindlimb musculature have not been evaluated.

Despite the growing number of studies in thermography, there are no thermographic studies to date that evaluate the effect of walking on temperature of muscles in dogs. This may have more clinical applications for companion animals compared to previous thermographic studies that focused on sporting and working dogs.

We propose to use thermography to measure changes in temperature of canine hindlimb muscles (gastrocnemius, gracilis, and biceps femoris muscles) after 6 min of leash walking. To the author's knowledge, the effects of walking on the temperature of canine hindlimb musculature have not been previously reported. A pilot study documenting changes in superficial muscle temperatures after light exercise could lead to further studies detecting early signs of muscle strain in dogs as demonstrated in human athletes (7).

The objective of the current study was to evaluate changes in temperature of hindlimb muscles before and after 6 min of leash walking in healthy dogs. Our hypothesis was that 6 min of walking will result in significant increases in temperature of hindlimb muscles during walking compared to baseline.

## Materials and Methods

### The Animals

This study was approved by the institutional animal care and use committee (IACUC) of the Animal Medical Center (AMC) of New York and the IACUC of the Animal Care and Control (ACC) of New York. Thirteen healthy Staffordshire terrier mixed breed dogs were prospectively evaluated for enrollment in this study. The study population included (10 dogs) recruited from the ACC and (1 dog) from owned pets at AMC. The owners of the dogs or the shelter coordinator (in the case of ACC dogs) were asked for consent prior to enrolment. All dogs underwent a physical exam and orthopedic exam (LXA, JR) and found to be clinically normal and had no history of orthopedic disease. Dogs with skin disease, shaving, or medium to long haired coats were excluded. Dogs with body condition scores <5 or >6 on a 9 point scale were also excluded (33). Dogs with any physical exam abnormalities (including any heart murmurs or arrhythmias) were excluded. Dogs were confined to a temperature-controlled environment (20°C) throughout the study. All physical exams were performed at least 30 min prior to thermal image capture to minimize the effect of handling on thermal patterns.

### Thermographic Imaging

**Figure 1:**
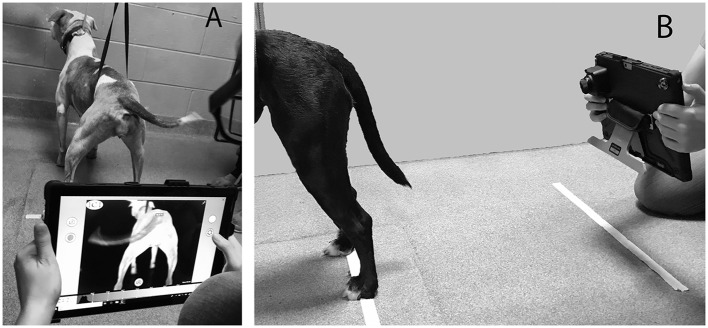
Demonstration of thermographic image capture method from caudal view **(A)** and lateral view **(B)**.

**Figure 2:**
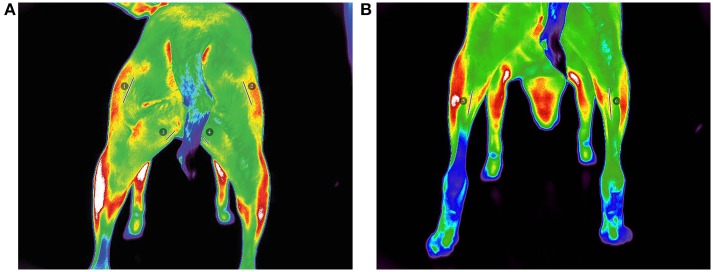
Pre-walk thermographic images demonstrating linear ROI selection of **(A)** (1) left biceps femoris, (2) right biceps femoris, (3) left gracilis, (4) right gracilis, **(B)** (5) left gastrocnemius, and (6) right gastrocnemius.

**Figure 3:**
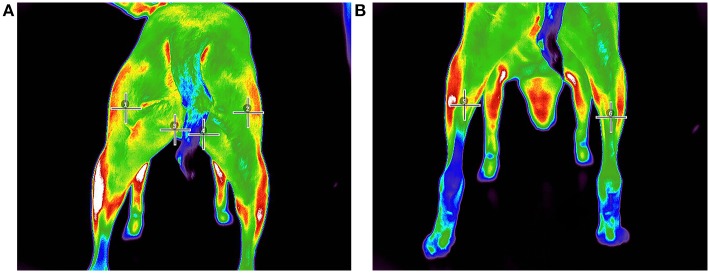
Pre-walk thermographic image demonstrating point ROI selection of **(A)** (1) left biceps femoris, (2) right biceps femoris, (3) left gracilis, (4) right gracilis, **(B)** (5) left gastrocnemius, and (6) right gastrocnemius.

All thermographic images were taken with a thermal camera with the resolution of 640X512^1^. Two thermographic images were obtained 24 inches from the caudal aspect of hindlimbs of each patient (one proximal and one distal) at ~90° using a tablet-based infrared sensing camera as described in a previous study [(23); Figures 1–3]. Dogs were then walked for 6 min on a pressure sensing walkway at a comfortable walking speed (0.9–2.1 m/s) as previously described (27, 28). At each end of the pressure sensing walkway, the dogs were turned such that the number of left and right turns were equal in each dog. Less than 1 minute after completing the 6MWT, the same 2 hindlimb thermal images were repeated for each subject as described above. There was no handling of the regions of interest within 30 min of image capture.

### Data Analysis

All images were analyzed with a thermal imaging software program^2^ using the medical color palette for viewing and analysis. All analyses were performed by the same investigator (JR). From the images, 3 superficial temperature points (PT) were selected: gastrocnemius, gracilis, and biceps femoris muscles as described in previous studies (21, 23). In an effort to reduce variability of region of interest (ROI) selection, a linear (LN) ROI on midline of the length of each muscle belly was selected for the gastrocnemius, gracilis, and biceps femoris muscles from which a mean temperature was calculated. The thermal differences were then extracted from the thermographic images by calculating the temperature of the hindlimb muscles before and after exercise.

Objective gait analysis was used to confirm a sound gait in the patient population. Objective gait analysis was collected in a quiet, temperature-controlled room using a temporospatial pressure sensing walkway^3^. The walkway system was equipped with a portable mat (4.3 × 2 m) containing 16,128 encapsulated sensors. The mat was calibrated by the manufacturer and raw data was captured by a computer equipped with software designed to analyze quadruped gait. Each dog was walked across the length of the gait analysis for the entire 6MWT duration. Walks were only included for data analysis if gait cycle velocities varied <10% and patient head carriage was forward facing. The first 3 successful walks per subject were pooled into analyzed data as performed in a previous study (34).

### Statistical Analysis

Baseline descriptive statistics are presented as mean and standard deviation for normally distributed variables whereas non-normally distributed variables are presented as median and range. Between groups, analyses of baseline variables were performed using analysis of variance (ANOVA) or the Wilcoxon test as appropriate for the data distribution within patient variation was determined by a dependent repeated measures ANOVA. The normality of the error residuals was analyzed by Kolmogorov-Smirnoff analysis and deemed normal. Analysis for proportions of categorical variables was performed using Chi-Square analysis or Fisher's exact test where appropriate. All analyses were considered significant if *p* < 0.05 and were carried out using a commercially available statistical software (SAS 9.4, SAS Institute Inc., Cary, North Carolina 2019).

## Results

### Dogs Included

Eleven healthy Staffordshire terrier mixed breed dogs met inclusion criteria and were enrolled. There were 3 neutered males, 2 intact females, 5 spayed females, and 1 intact male with weights and ages ranging from 17.5 to 37.3 kg (mean 23.3 ± 5.6 kg) and 2 to 7 years (mean 4 ±1.9 years), respectively.

### Objective Gait Analysis

Data obtained through pressure sensing walkway demonstrated an average forelimb total pressure index (TPI) of 29.1 ± 3.2 and hindlimbs TPI of 20.9 ± 3.2. TPI symmetry ratios (left hind:right hind) ranged from 0.87 to 1.13.

### Thermography

#### Changes After Exercise

There were no significant differences in superficial temperatures of the gastrocnemius, gracilis muscles, and biceps femoris after 6MWT compared to baseline using the point selection method for ROI (Table 1). The linear (LN) ROI selection of the biceps femoris demonstrated significantly increased temperature exercise for both the left and the right sides (*p* = 0.003 and *p* < 0.0001, respectively). There was also a significant increase in temperature of the LN gracilis muscle when left and right sides were averaged together (*p* = 0.032). While mean LN gracilis temperatures were both increased when left and right sides were evaluated independently, this difference was not significant (*p* = 0.083, *p* = 0.174, respectively).

**Table 1:** Overall ± SD as well as minimum (min) and maximum (max) superficial temperatures obtained from thermographic images.

**Pre walk**						**Post walk**				
**Muscle**	**Side**	**Mean**	**SD**	**Min**	**Max**	**Mean**	**SD**	**Min**	**Max**	***P*-Value**
GST	Left	33.99	1.74	30.90	36.30	34.24	1.27	32.40	35.80	0.587
	Right	33.25	1.65	29.80	35.30	33.58	1.39	31.00	35.60	0.374
	Average	33.62	1.70	29.80	36.30	33.91	1.34	31.00	35.80	0.273
GST_LN	Left	33.61	1.25	31.50	35.30	33.78	1.13	31.60	35.00	0.580
	Right	33.42	1.34	31.00	35.40	33.58	1.19	31.30	35.10	0.600
	Average	33.51	1.27	31.00	35.40	33.68	1.14	31.30	35.10	0.448
GRC	Left	34.77	1.17	32.80	36.00	34.95	1.04	32.90	36.50	0.474
	Right	34.90	1.06	32.80	36.20	35.05	1.09	32.80	36.90	0.521
	Average	34.84	1.09	32.80	36.20	35.00	1.04	32.80	36.90	0.339
GRC_LN	Left	33.74	1.52	31.10	35.30	34.26	1.25	31.90	35.90	0.083
	Right	34.24	1.46	31.20	35.70	34.65	0.98	32.60	36.00	0.174
	Average	33.99	1.47	31.10	35.70	34.45	1.11	31.90	36.00	0.032
BF	Left	32.22	1.34	29.50	33.80	32.46	1.37	30.00	34.10	0.501
	Right	32.21	1.48	29.60	34.10	32.45	1.21	30.70	34.30	0.516
	Average	32.21	1.38	29.50	34.10	32.45	1.27	30.00	34.30	0.351
BF_LN	Left	32.54	1.04	30.60	34.10	33.36	1.06	31.90	35.00	0.004
	Right	33.13	1.15	30.80	34.60	34.63	1.18	32.40	36.50	<0.0001
	Average	32.83	1.11	30.60	34.60	34.00	1.27	31.90	36.50	<0.0001

*Measurement for point selected left and right legs of gastrocnemius (GST), gracilis (GRC), and biceps femoris (BF) and point linear selected gastrocnemius (GST_LN), gracilis (GRC_LN), and biceps femoris (BF_LN) before and after walking*.

#### Effect of Region of Interest on Standard Deviation

The overall (left and right limbs pooled) standard deviation of point selected values were greater than LN selected values of the biceps femoris (1.35 and 1.11) and gastrocnemius (1.51 and 1.23). In contrast, standard deviation for the gracilis measurements were decreased using point selection vs. LN selection (1.09 and 1.3).

#### Differences Between Sides

The difference between left and right LN biceps femoris was statistically significant pre-exercise (32.5 and 33.1°C, respectively, *p* = 0.03) and post exercise (33.4 and 34.6°C, respectively, *p* < 0.0001). There was no significant difference between sides for both gracilis and gastrocnemius muscles using either ROI method, at any point in time.

## Discussion

### Thermography

After a 6MWT, there was no significant difference between mean pre and post superficial temperatures of biceps femoris, gastrocnemius, and gracilis when using the point ROI method employed by previous studies (21, 23). However, when the linear ROI method was used, there was a statistically significant temperature increase in biceps femoris and gracilis muscles post exercise. Thus, we accepted our hypothesis that the temperature increase was significant in the biceps femoris and gracilis muscles pre and post 6MWT, but was rejected for the gastrocnemius muscle. This temperature increase could correspond to the increased work during walking exerted by the biceps femoris and gracilis muscles.

The accuracy of visual selection of muscles based on thermal imaging has not been assessed. ROI interest selection has been variable among thermography studies in canines. Visual point-based selection as used previously in evaluation of hindlimb musculature (21, 23) is subjective when muscle groups have not been clearly demarcated. In an effort to reduce variability in point selection, we employed a linear method of ROI selection as described in a previous study on humans with knee osteoarthritis (35). The overall decreased variability and significant increase in temperatures using this LN method may reflect that this is a more reliable form of ROI selection. In a previous study comparing dogs with normal and dysplastic elbows by McGowan et al. (15) pattern analysis classified abnormal joints 100% of the time whereas the temperature differences in the ROI were small. This suggests pattern analysis rather than regional temperature differences may produce more consistent results. Further study on ROI selection and pattern analysis is necessary to determine which screening method yields the most accurate results.

An alternative method of isolating a ROI would be through selection of a muscle group using a free hand manual outlining of the muscle belly or placement of visual markers on patients based on anatomical landmarks. Even with such methods, however, muscle isolation is not 100% reliable due to summation effect with the overlying tensor fascia latae and potential increase in surface temperatures. Therefore, such methods may be unreliable and impractical in a clinical setting.

A previous study demonstrated increases in temperature of the biceps femoris only in military working dogs <7 months old exercised on a treadmill for 12 min (21). In another study (23), the gastrocnemius muscle temperature was most elevated in greyhounds after intense racing activity. In both studies, the animals were preconditioned and undergoing regular training. The subjects used in the current study were kenneled with scheduled twice daily leashed walks and otherwise not part of any athletic training program. It is unknown how these conditioning differences would affect the results of this study; however, a study in humans comparing temperatures in trained vs. untrained exercising humans demonstrated greater temperature increases in trained individuals after exercise (36). In contrast, in this study there was a decrease in the maximum values of the gastrocnemius after exercise (with exception of the right side with point selection). This may reflect a temporary post exercise decrease in surface temperature as described in humans (37). However, a component of that temperature decrease may be due to evaporative cooling that is not present in dogs.

Interestingly, previous studies with conditioned dogs (21, 23) did not report significant change in temperature of the gracilis muscles after exercise as found in the current study using the linear ROI selection method. This difference could demonstrate increased sensitivity of using a linear ROI for assessment of the gracilis muscle. This has clinically relevant applications in both sporting and working dogs. Greyhounds are reported to be susceptible to gracilis muscle tears and German shepherds are commonly afflicted with gracilis fibrotic myopathy (38). Perhaps this method of ROI selection could be used for early detection of pathology and subsequent injury prevention.

Clinically, it is useful to establish expected changes in hindlimb temperature occurring after various levels of activity. Six minutes of walking is considered relatively light aerobic exercise and may reasonably occur prior to examination in a clinic or as part of a warm up in competition. This data represents expected temperature variation that could occur under those circumstances. A human study (39) reported light aerobic exercise leads to decreases in temperature whereas intense anaerobic exercise increases temperature until failure in the biceps brachii. There were several dogs in the present study with decreased muscle temperatures post walk and this perhaps may be a reflection of this phenomenon. Further study focused on exercise transitions from aerobic to anaerobic exercise is needed to determine if these temperature changes exist in dogs.

The increased temperature observed in the biceps femoris compared to other hindlimb musculature in the present study may be explained by its unique function during the gait cycle. The biceps femoris is composed of cranial and caudal parts that are both responsible for protraction of the hindlimb and flexion of the stifle, respectively. A previous study observed biceps femoris muscle activity using electromyography (EMG) in both swing and stance phase in dogs trotted on a treadmill (40). In another study, EMG in dogs showed muscular activity in the biceps femoris that was consistent with elastic storage of kinetic energy end of swing phase and recovery during propulsive stroke in the tibial portion of active muscle (41). Activity of the gastrocnemius muscle during stretch and shortening was only observed at faster trotting speeds and a gallop. This may explain the lack of temperature changes observed in gastrocnemius in the current study and supports the results from a previous study that showed increased temperatures in the gastrocnemius in post-racing greyhounds (23).

One of the challenges of utilizing thermography in practice is determining the clinical significance of results. Thermography studies in humans have established elevations in temperatures of muscles caused by local inflammation and vasodilation. Increases in temperature have correlated with increases in muscle volume of the biceps femoris in one study (37). Other studies have indicated a correlation of surface temperature with delayed onset of muscle soreness (7). Previous reports in horses and humans have determined that asymmetry of >1°C is indicative of underlying pathology (42). A more recent study in humans (43), found variations of 0.5 ± 0.3°C in healthy subjects. However, these studies used data from broader, box-shaped ROIs. In a previous study (23), racing greyhounds had differences between right and left limbs ranging from 0 to 4°C that were not clinically nor statistically significant. The present study showed statistically significant temperature asymmetries up to 3°C in the same muscle groups on opposite sides in healthy subjects. This is in contrast to the study by Loughin and Marino (12) that found no significant difference between sides in dogs. However, the Loughin study used a broader method (box-shaped) for ROI selection and thus cannot be directly compared. It is also important to note the sensitivity of the infrared camera used in this study is higher compared to previous studies (0.02°C compared to 0.05°C) which would be expected to decrease variability. These differences may represent normal variation in dogs or potentially secondary to cooling of superficial skin temperatures on the dependent side on which the animal was laying prior to image capture.

### Objective Gait Analysis

The results of objective gait analysis showed hindlimb TPI symmetry ratios (left hind:right hind) ranging from 0.87 to 1.13. This was consistent with the results of previous studies in a population of normal dogs (34) which reported hindlimb symmetry ratio of 1 ± 0.12.

### Limitations

Limitations of this study include the small sample size and homogenous population of dogs. Selection of dogs with different coat lengths and body structure may have yielded different results. However, this Staffordshire terrier mixes were chosen in this study for the short coat characteristic of the breed thereby decreasing temperature variance noted in previous studies secondary to coat length (13, 44). In addition, coat color was not consistent; however, this has not shown to affect thermal images (45).

Selection of ROI requires manual demarcation which is likely subject to individual variation. While every attempt was made to standardize the acquisition of thermography images, the analysis may have limited accuracy from changes in posture, camera angle, intra-observer variability, and inter-observer variability. Indeed, it is possible that our findings were not due to increases in temperature, but rather these aforementioned methodologic issues. Future studies assessing the accuracy and precision of thermography are required to validate our findings. ROI in this study were selected by a single observer and therefore intraobserver and interobserver variation were not assessed. Box region of interest (ROI) selection as used in other studies is less practical in the context of this study due to the natural shape of hindlimb musculature in the images captured. A line ROI selection method was chosen in this study as it more closely follows the shape of the studied hindlimb musculature. Additionally, the observer in this study was not blinded to the status of the animal in the image observed potentially leading to biased point selection. Further studies are required to evaluate the impact of interobserver variability.

While most dogs maintained a comfortable walking gait during the 6MWT, some of the dogs occasionally converted into a trot as a preferred gait. It is unknown how much this change in gait influenced the results obtained.

This study evaluated post exercise temperatures at a single time point (<1 min after 6MWT) and it is possible that this initial increase in temperature may be subsequently followed by a peak in temperature as in human studies (37). The focus of this study was to evaluate muscle temperatures immediately post light aerobic exercise as compared to increases after high intensity exercise in other studies (21, 23) and additional studies are required to determine when peak temperatures occur.

## Conclusion

The biceps femoris and gracilis muscles showed statistically significant increases in superficial temperatures after 6 min of walking when using linear region of interest selection method. There were no significant changes in temperature of gastrocnemius muscles using both linear and point ROI methods. Differences in right and left muscle temperatures may be found in healthy individuals. Measurement of numerous points along the entire length of the biceps femoris and gastrocnemius muscles may provide a more accurate assessment of the increased vascularity within the tissues resulting from work compared to single point selection. Region of interest selection method should be considered when interpreting thermography results.

## Data Availability Statement

All datasets generated for this study are included in the article/supplementary material.

## Ethics Statement

The animal study was reviewed and approved by Institutional animal care and use committee (IACUC) of the Animal Medical Center (AMC) of New York and the IACUC of the Animal Care and Control (ACC) of New York. Written informed consent was obtained from the owners for the participation of their animals in this study.

## Author Contributions

LA and JR developed conception of the study and study protocols and participated in data collection. JR was responsible for analysis of the data. The manuscript was written by JR and LA and was reviewed and edited by all authors. Statistical analysis was performed by KL. RG provided expert advice in data interpretation and analysis.

## Conflict of Interest

KL was employed by Lamb Statistical Consulting and Scientific Writing LLC. RG was employed by Sportsvet Veterinary Consulting Services. The remaining authors declare that the research was conducted in the absence of any commercial or financial relationships that could be construed as a potential conflict of interest.

## Footnotes

^1^Digitherm IR Tablet 640 Thermal Imaging DTI System 640X512 Sensor: 327,680 (pixels) points of temperature measurement, ±1°C Accuracy, <0.02°C Sensitivity Calibration, Ocala, FL.

^2^IR Flash Pro.

^3^Gait4Dog Walkway, Sparta, NJ.

## Abbreviations

BF: Biceps femoris muscle
GRC: Gracilis muscle
GST: Gastrocnemius muscle
LN: Measurements using linear region of interest selection method
PT: Measurements using point region of interest selection method
ROI: Region of interest
6MWT: 6 min walk test
TPI: Total pressure index.
